# Somatostatin Receptor Based Imaging and Radionuclide Therapy

**DOI:** 10.1155/2015/917968

**Published:** 2015-03-24

**Authors:** Caiyun Xu, Hong Zhang

**Affiliations:** ^1^Department of Nuclear Medicine, The Second Affiliated Hospital of Zhejiang University School of Medicine, Hangzhou, Zhejiang 310009, China; ^2^Zhejiang University Medical PET Center, Zhejiang University, Hangzhou, Zhejiang 310009, China; ^3^Institute of Nuclear Medicine and Molecular Imaging, Zhejiang University, Hangzhou, Zhejiang 310009, China; ^4^Key Laboratory of Medical Molecular Imaging of Zhejiang Province, Hangzhou, Zhejiang 310009, China

## Abstract

Somatostatin (SST) receptors (SSTRs) belong to the typical 7-transmembrane domain family of G-protein-coupled receptors. Five distinct subtypes (termed SSTR1-5) have been identified, with SSTR2 showing the highest affinity for natural SST and synthetic SST analogs. Most neuroendocrine tumors (NETs) have high expression levels of SSTRs, which opens the possibility for tumor imaging and therapy with radiolabeled SST analogs. A number of tracers have been developed for the diagnosis, staging, and treatment of NETs with impressive results, which facilitates the applications of human SSTR subtype 2 (hSSTr2) reporter gene based imaging and therapy in SSTR negative or weakly positive tumors to provide a novel approach for the management of tumors. The hSSTr2 gene can act as not only a reporter gene for *in vivo* imaging, but also a therapeutic gene for local radionuclide therapy. Even a second therapeutic gene can be transfected into the same tumor cells together with hSSTr2 reporter gene to obtain a synergistic therapeutic effect. However, additional preclinical and especially translational and clinical researches are needed to confirm the value of hSSTr2 reporter gene based imaging and therapy in tumors.

## 1. Introduction

Somatostatin receptors belong to the typical 7-transmembrane domain family of G-protein-coupled receptors [1]. Five distinct subtypes (termed SSTR1-5) have been identified, with SSTR2 showing the highest affinity for natural SST and synthetic SST analogs [2]. Most NETs, including pituitary adenoma, gastroenteropancreatic tumor (GEP-NET), pheochromocytoma, neuroblastoma, paraganglioma [3, 4], medulloblastoma [5], and medullary thyroid carcinoma [6], have relatively high expression levels of SSTRs, which opens the possibility for tumor imaging and therapy with radiolabeled SST analogs. A number of tracers have been produced due to encouraging initial results from the applications of radiolabeled ligand-receptor systems [7, 8]. The most commonly used radiopharmaceutical for somatostatin receptor scintigraphy (SRS) is [^111^In-DTPA^0^]octreotide, which has proven its role in the diagnosis and staging of NETs [9]. Favorable results also have been observed in NET imaging using other *γ*-emitting tracers like ^99m^Tc-Depreotide and [^111^In-DOTA]lanreotide [10, 11]. More recently, positron-emitting radiotracers have been developed and seem to be more promising. ^68^Ga-DOTA-peptides used for positron emission tomography (PET) or positron emission tomography/computed tomography (PET/CT) imaging of NETs have been reported by various research groups with higher sensitivity and specificity compared to SRS and conventional imaging modalities [12–14]. In addition, SST analogs labeled with therapeutic radionuclides, such as ^111^In, ^90^Y, ^177^Lu, and ^213^Bi, have been applied in peptide receptor radionuclide therapy (PRRT) for patients with inoperable and/or metastatic NETs [15]. The results that were obtained with [^90^Y-DOTA^0^-Tyr^3^]octreotide (^90^Y-DOTATOC) and [^177^Lu-DOTA^0^-Tyr^3^]octreotate (^177^Lu-DOTATATE) are particularly promising in terms of tumor regression, overall survival, and quality of life, if adequate renal protection is used [16, 17].

However, SSTR based imaging and therapy are only available in SSTR positive tumors. Many malignant human cancers were poorly or not detected to express SSTR subtypes [18, 19]. Surgery, chemotherapy, and radiotherapy have limited effects on improving survival for patients diagnosed with these cancers [20]. Therefore, some studies [21, 22] have tried to explore the applications of hSSTr2 reporter gene based imaging and therapy in SSTR negative or weakly positive tumors to provide a new strategy for the management of these tumors. To this end, the target gene hSSTr2 should be inserted into gene transfer vectors to induce transfected tumors to express SSTR2 [23–25] and assure that hSSTr2 expression could occur on tumor cell membranes. Optical imaging and biopsy have been taken to identify whether gene transfer succeeds or not. However, poor tissue penetration of light-based reporter systems, such as green fluorescent protein [23, 26] and luciferase [27], hampers a comprehensive assessment of whole organism or targeted tumors [20]. On the other hand, biopsy is an invasive technique and can only evaluate the outcome of gene expression [28]. Nuclear imaging can avoid the unclear images in deep tissues and the need of tissue sampling for histological analysis of gene expression. Tumors transfected with hSSTr2 gene can be monitored by external gamma camera, single photon computed emission tomography (SPECT), or PET imaging techniques with radiolabeled SST analogs [29]. These* in vivo* hSSTr2 based imaging methods have several advantages: repetitively observing the expression levels of hSSTr2 or other genes within transfected tumors [30], studying the biodistribution of gene transfer vectors [25], predicting the effects of PRRT, and monitoring the time course of tumor growth and the efficacy of antitumor gene therapy [21]. The hSSTr2 gene transfer not only allows for some SSTR negative tumors to be imaged* in vivo*, but also can be useful for antitumor radionuclide therapy. SST analogs labeled with therapeutic radionuclides can be delivered to the cell receptor targeting site and are able to induce the internalization of ligand-receptor complexes [31–33]. The trapped radiopeptides in transfected tumor cells have been considered to be necessary for internal local irradiation, which offers an alternative approach to conventional therapeutics for SSTR negative tumors [22]. Furthermore, by the simultaneous inclusion of a second therapeutic gene like thymidine kinase (TK) or cytosine deaminase (CD), it is possible to obtain a dual gene vector that includes hSSTr2 working as a reporter gene for* in vivo* imaging as well as a therapeutic gene for radionuclide therapy. Preliminary studies suggested that a synergistic therapeutic effect could be achieved following dual gene transfer with one vector encoding both hSSTr2 reporter gene and a second therapeutic gene [21, 34].

In this review, we summarize the performances of SSTR based imaging and radionuclide therapy in NETs and introduce the applications of hSSTr2 reporter gene based imaging and therapy with radiolabeled SST analogs in SSTR negative or weakly positive tumors.

## 2. Somatostatin Receptor Based Imaging

Computed tomography (CT) and magnetic resonance imaging (MRI) are commonly used to detect NETs and have sensitivity between 50 and 80% based on anatomic characteristics [37]. In comparison, functional imaging modalities, that is, PET, SPECT, or scintigraphy, have shown higher sensitivity and specificity in visualizing primary tumors and their metastases. During the past two decades, SRS has been widely used for the diagnosis and staging of NETs. [^123^I, Tyr^3^]octreotide was the first radiolabeled SST analog utilized for* in vivo* imaging [38]. Unfortunately, high nonspecific accumulation was observed in the liver and intestine, which has limited its ability to locate early primary tumors [39]. Soon a new radiotracer consisting of octreotide, the chelator DTPA, and the radionuclide ^111^In was developed as [^111^In-DTPA^0^]octreotide (OctreoScan). It has been approved by the Food and Drug Administration and was commercially available as ^111^In-pentetreotide [40]. An early study [41] tried to evaluate the potential of [^111^In-DTPA^0^]octreotide in the visualization of NETs. Patients (*n* = 6) with proven tumors (three carcinoids, three insulinomas) were scanned using both [^111^In-DTPA^0^]octreotide and [^123^I, Tyr^3^]octreotide, obtaining the same results in 4 patients. Nevertheless, [^111^In-DTPA^0^]octreotide images showed higher resolution at 21 hours after injection with a more satisfactory tumor-background ratio. Several papers [42–44] reported high sensitivity, varying between 67 and 100%, in NET imaging with [^111^In-DTPA^0^]octreotide. In the management of patients with NETs, [^111^In-DTPA^0^]octreotide scintigraphy can be used not only to detect primary NETs and their metastases, but also to follow up patients with known tumors, monitor tumor response to therapy, and predict the efficacy of PRRT for patients with inoperable and/or metastatic NETs [41, 45, 46]. These good qualities lay the foundation for [^111^In-DTPA^0^]octreotide becoming the gold standard for NET imaging [40]. From 2012, SPECT/CT scanning using [^111^In-DTPA^0^]octreotide is an important part of the diagnostic work-up of patients with NETs in the consensus guidelines of European Neuroendocrine Tumor Society [47, 48]. However, some NETs, like primary sympathetic paragangliomas, show low uptake of [^111^In-DTPA^0^]octreotide in tumor regions [49] while other NETs may become OctreoScan negative with time due to tumor dedifferentiation [10]. Efforts are therefore spent on developing radiolabeled SST analogs to be capable of imaging SSTR positive tumors with higher sensitivity, specificity, and accuracy. The ^99m^Tc-labeled agent, ^99m^Tc-Depreotide, has received regulatory approval in the United States and Europe for use in the detection of lung cancer [50]. It binds to a wide range of SSTR subtypes (SSTR2, SSTR3, and SSTR5) and has shown promise in diagnosing a variety of tumor types [51–53], including some OctreoScan negative NETs [10]. ^111^In-DOTA-lanreotide is another *γ*-emitting tracer with different affinity for SSTR subtypes compared to [^111^In-DTPA^0^]octreotide. Tumors expressing mainly SSTR3 and/or SSTR4, for example, intestinal adenocarcinomas, may be well visualized by ^111^In-DOTA-lanreotide [11, 54].

**Figure 1:**
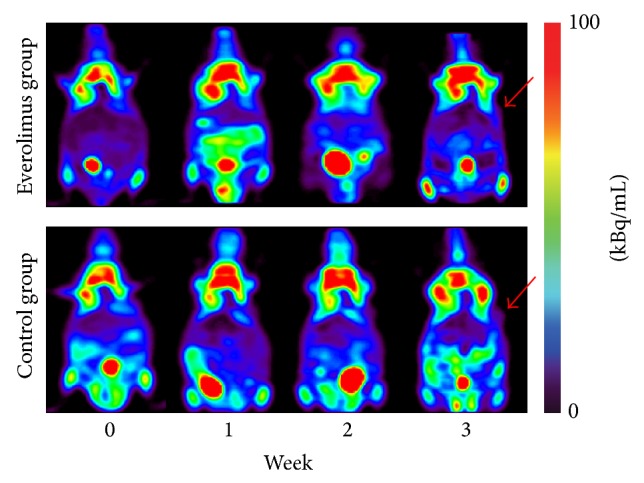
Serial PET images demonstrating glucose metabolism changes before (week 0) and after everolimus treatment (week 1, week 2, and week 3) in nude mice bearing Bon-1 pancreatic tumor xenografts (red arrows). The two groups are presented as the everolimus treatment group and the control group. Images are shown in axial view. No significant difference of the tumor uptake was found between the two groups at each time point after everolimus treatment.

With the emerging of PET scanning, a variety of positron-emitting tracers have been produced. [^18^F]fluorodeoxyglucose (^18^F-FDG) is the most widely used radiotracer for tumor staging and treatment response surveillance in a number of tumor types. The trapped ^18^F-FDG in cells can reflect glucose metabolism profile of normal tissues and lesions [40]. In general, malignant tumors demonstrate increased uptake of ^18^F-FDG and can be distinguished from normal tissues. It has been demonstrated in patients affected by NET that ^18^F-FDG PET has a high accuracy for poorly differentiated tumors [55, 56]. However, ^18^F-FDG is not indicated primarily for NET imaging since most NETs present low proliferative activity and well differentiation. We attempted to use ^18^F-FDG imaging to monitor everolimus effect on tumor growth in Bon-1 pancreatic NETs. The results showed that* in vivo* tumor volumes measured relative to baseline were significantly lower in the everolimus group compared to the control group, whereas the uptake of ^18^F-FDG was quite low in tumor regions and showed no significant difference between the two groups at any time point after everolimus treatment (Figure 1).

**Figure 2:**
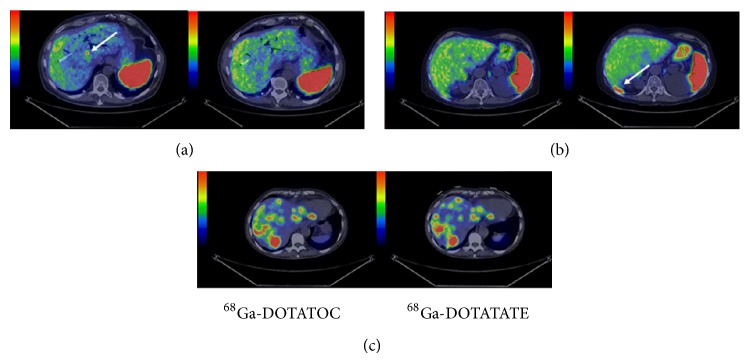
Transaxial images of liver metastases demonstrating cases of higher detection rate for ^68^Ga-DOTATOC ((a): patient 6, PET/CT fusion); higher detection rate for ^68^Ga-DOTATATE ((b): patient 8, PET/CT fusion); and equal detection rate ((c): patient 1, PET/CT fusion). Whole-body scans were conducted at 1 hour after injection. Arrows point toward hepatic metastases [35].

**Figure 3:**
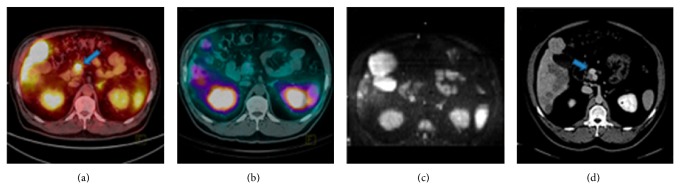
A 49-year-old man with NET of unknown origin for over 4 years. ^68^Ga-DOTATATE PET/CT (a) identified primary pancreatic lesion (arrow), whereas SPECT/CT (b) and WB DWI (c) did not. This lesion was noted only retrospectively (arrow) on dedicated abdominal CT (d) performed 4 years previously [13].

Positron-emitter ^68^Ga can be produced just depending on ^68^Ge-^68^Ga generator, so it is available in departments without a cyclotron. ^68^Ga-labeled SST analogs with a quite short half-life (68 min) have exhibited great potential for PET imaging of NETs and their metastases [12, 42, 57]. Some [38, 58] predicted that ^68^Ga-labeled peptides are the most likely candidates for such a universal tracer applied in the diagnosis, staging, and restaging of patients with NETs instead of ^111^In-DTPA-octreotide. [^68^Ga-DOTA^0^-Tyr^3^]octreotide (^68^Ga-DOTATOC), [^68^Ga-DOTA^0^,1NaI^3^]octreotide (^68^Ga-DOTANOC), and [^68^Ga-DOTA^0^-Tyr^3^]octreotate (^68^Ga-DOTATATE) are three main ^68^Ga-labeled SST analogs widely utilized to NET imaging and patient selection for PRRT [59]. They demonstrate slightly different affinities for the five SSTR subtypes. ^68^Ga-DOTATATE is SSTR2 selective, presenting 10-fold higher affinity for SSTR2* in vitro* than that of ^68^Ga-DOTATOC [60], which has high affinity for SSTR2 and SSTR5. In comparison, ^68^Ga-DOTANOC has a wider receptor binding profile, able to specifically bind to SSTR2, SSTR3, and SSTR5 [61]. These differences may affect their efficiency in the detection of NET lesions. A study [60] explored 40 patients with metastatic NETs, who underwent both ^68^Ga-DOTATOC and ^68^Ga-DOTATATE PET/CT. The diagnostic accuracy was almost the same between the two ^68^Ga-DOTA-conjugated peptides. However, tumor uptake varied considerably both within and between patients. Eighteen patients displayed only lesions with higher uptake of ^68^Ga-DOTATOC than ^68^Ga-DOTATATE and the reverse situation was found in 4 patients. The other 18 patients displayed a mixture of lesions with higher uptake of either ^68^Ga-DOTATATE or ^68^Ga-DOTATOC. These differences in tumor uptake of the two radiopeptides were also reported by a latest study [35] (Figure 2). The possible reasons for such a variation could be the extensive difference in the SSTR subtype profile and the nonstandardized examination conditions. For tumors expressing broader SSTR subtypes, ^68^Ga-DOTANOC may be more efficient to detect NET lesions; Wild et al. conducted a study [61] in which 18 patients with clearly diagnostic GEP-NETs were imaged with ^68^Ga-DOTANOC and ^68^Ga-DOTATATE. Consequently, the SSTR2, 3, 5 specific radiotracer ^68^Ga-DOTANOC detected significantly more lesions than the SSTR2 selective radiotracer ^68^Ga-DOTATATE. Although the diagnostic efficacy varies among the three radiopeptides, PET imaging with ^68^Ga-DOTA-conjugated peptides offers higher sensitivity and specificity compared with SRS and conventional imaging modalities. In an early study [12], ^68^Ga-DOTATOC PET was compared with SRS and CT in the visualization of known or suspected NETs (*n* = 84 patients). As a consequence, ^68^Ga-DOTATOC PET had a significantly higher diagnostic efficacy than SRS and CT in various clinical situations (initial diagnosis, staging, and follow-up), which have affected clinical management in a considerable number of patients, especially when compared with CT. A latest study [13] aimed to compare NET lesion detectability among SPECT/CT, ^68^Ga-DOTATATE PET/CT, and whole-body diffusion-weighted MR imaging (WB DWI). The results showed that ^68^Ga-DOTATATE PET/CT seemed to be more sensitive for detection of NET lesions, especially for bone and unknown primary lesions (Figure 3). Comparison of ^68^Ga-DOTANOC PET/CT and conventional imaging (mainly CT and MRI) was undertaken in a clinical study [14]. Conventional imaging was available in included patients (*n* = 111) with 93 patients suspected of NETs; ^68^Ga-DOTANOC PET/CT was superior for detection of NETs with high sensitivity, specificity, positive predictive value, negative predictive value, and accuracy.

NETs were formerly described as APUDomas (amine precursor uptake and decarboxylation). Amine precursor such as 5-hydroxy-L-tryptophan (5-HTP) and L-dihydroxyphenylalanine (L-DOPA) may be absorbed into tumor cells and turned into their corresponding amines, dopamine and serotonin. Based on these characteristics of APUD system, ^11^C-labelled and ^18^F-labelled L-DOPA (^11^C-L-DOPA, ^18^F-L-DOPA) as well as 5-HTP (^11^C-5-HTP, ^18^F-5-HTP) have been developed to visualize NETs [62–64]. Their imaging performances are quite good and they can provide additional information for the diagnosis, staging, and management of NETs. One shortage is that nonfunctioning NETs cannot be detected using these tracers. Now they are employed as problem solving tools when other imaging technique results are negative or contradictory [29].

## 3. Somatostatin Receptor Based Therapy

Surgical treatment suffices for the majority of NETs, but malignant, recurrent, and metastatic tumors need further treatment in order to gain a lengthening of time to progression [65]. Systemic chemotherapy is currently used for patients with poorly differentiated NETs, whereas tumor response is difficult to be assessed as these tumors are not highly chemosensitive and spontaneous standstill or regression is noticed in the time course of tumor growth [66]. A limited number of studies have explored the role for the therapeutic use of external beam radiation therapy in malignant NETs [67]. SST analogs, predominantly octreotide and octreotate, suppressing hormone production, have improved symptoms as well as prognosis in tumors. But the employment of SST analogs must be weighed against the tachyphylaxis and the limited antitumor effects [68]. PRRT using radiolabeled SST analogs has proven to be an effective therapeutic option for NET patients with inoperable and/or metastasized diseases. SST analogs labeled with therapeutic radionuclides, such as ^111^In, ^90^Y, ^177^Lu, and ^213^Bi, are able to specifically bind to SSTRs on tumor cells and deliver an effective radiation dose to tumors with minimal damage to normal tissues [69].

### 3.1. Studies with [^111^In-DTPA^0^]octreotide

^111^In not only emits *γ*-radiation, which penetrates tissues easily and can be imaged by a *γ*-scanner, but also emits therapeutic Auger and conversion electrons that play an antiproliferative role in malignant tumors with a short to medium tissue penetration [70]. Initial therapeutic studies [71, 72] performed with high radioactivity doses of [^111^In-DTPA^0^]octreotide in patients with metastatic NETs resulted in significant symptom relief but relatively few and short-lived objective tumor responses. These results are not unexpected since [^111^In-DTPA^0^]octreotide is not an ideal option for PRRT due to their small particle range of Auger electrons [73]. It has been recommended that SST analogs labeled with higher energy *β*-emitters, which, in reality, have obtained better response rates in various studies [74, 75], should be employed to treat SSTR positive tumors.

### 3.2. Studies with [^90^Y-DOTA^0^, Tyr^3^]octreotide

^90^Yttrium (^90^Y) is a *β*-particle emitter with a maximum energy of 2.3 MeV and a maximum range of 12 mm in tissue [76]. It is combined with a more stable chelator DOTA instead of DTPA and a modified SST analog octreotide to get a conjunction ^90^Y-DOTATOC. This tracer has superior therapeutic efficacy since adequate dose of radiation can be delivered to tumors, especially larger tumor masses, to cause cell damage [77]. After 15 years of experience, PRRT with ^90^Y-DOTATOC is generally well tolerated [78].

Forrer et al. selected 116 patients with metastatic NETs, who underwent PRRT with ^90^Y-DOTATOC (5994–7400 MBq/m^2^ body surface). All cases were positive in the scintigraphy. After the last administration, each patient was evaluated with respect to the therapeutic effects on tumor size. The objective response rate was found in 31 patients (26%), including 4% complete remission (CR) and 22% partial remission (PR). 72 patients (62%) showed stabilization of their diseases and the remaining patients (11%) were still progressive (Table 1). No serious side effects occurred and the toxicity was well tolerated [79]. A similar tumor response rate (24%, 2% of which were CR and 22% were PR) was found in a phase II study of 41 patients with GEP-NETs and bronchial tumors who were given intravenously four injections of a total of 6000 MBq ^90^Y-DOTATOC (Table 1). Grade III pancytopenia was the most severe adverse event occurring in 5% patients [80]. It is not possible to state that ^90^Y-DOTATOC is of great use for the management of inoperable and/or metastatic NETs when drawing conclusion from a relatively small sample. A study [84] conducted in a larger group of patients with a wide spectrum of NETs supplied more meaningful results. 1109 patients from 29 countries were treated with repeated cycles of ^90^Y-DOTATOC. Morphologic response was found in 378 patients (34.1%) and stable disease (SD) in 58 patients (5.2%). The median survival from diagnosis was 94.6 months, which was longer than the expected 33-month survival. Longer survival was associated with morphologic, biochemical, and clinical response as well as high tumor uptake in pretherapeutic SRS. Among all the patients, 143 were subjected to severe hematologic toxicities and 102 to permanent renal toxicity.

Except mentioned studies, there are a large number of reported articles assessing the therapeutic effects of ^90^Y-DOTATOC. Despite differences in protocols, the objective tumor responses in most of the studies with ^90^Y-DOTATOC are in the same range, approximately 20–28% in patients with NETs and for patients with GEP-NETs in the range of 28–38% [85].

### 3.3. Studies with [^177^Lu-DOTA^0^-Tyr^3^]octreotate

^177^Lutetium (^177^Lu) is a median energy *β*-emitter (0.5 MeV) with small particle range [86]. This allows for higher radiation dose delivered to smaller tumors and less damage to surrounding tissues than the radionuclide ^90^Y [87]. ^177^Lu also emits *γ* rays; thus ^177^Lu-labeled peptides can be used for treatment as well as for dosimetry and monitoring of tumor response. [DOTA^0^, Tyr^3^]octreotate (DOTATATE), which presents a ninefold increase in the affinity for SSTR2 compared with [DOTA^0^, Tyr^3^]octreotide [88], usually labeled with the radionuclide ^177^Lu, has been widely used in PRRT. The results that were obtained with ^177^Lu-DOTATATE are impressive in terms of tumor suppression and patient survival [17, 89].

An early clinical study [75] compared the therapeutic effects of ^177^Lu-DOTATATE with [^111^In-DTPA^0^]octreotide in 6 patients with SSTR positive tumors. After 24 hours, the uptake of ^177^Lu-DOTATATE was almost equal to that of [^111^In-DTPA^0^]octreotide for kidneys but was three- to fourfold higher for 4 of the analyzed tumors. Thus, ^177^Lu-DOTATATE potentially represents an important improvement since the higher radiation doses can be delivered to tumors with about equal doses to dose-limiting organs, especially kidneys. Latest data illustrated that, even with low radiation doses, ^177^Lu-DOTATATE could have antitumor effects in advanced pancreatic NETs [82]. Fifty-two patients were assigned to the following two groups: full dosage (FD) group (25.5 GBq, *n* = 26) and reduced dosage (RD) group (17.8 GBq, *n* = 26). Both groups showed antitumor activity, with 12% CR, 27% PR, and 46% SD in the FD group (Table 1), while 4% CR, 15% PR, and 58% SD in the RD group. Although response rate was higher in FD, no significant difference was found. However, progression-free survival was significantly longer after injection of a total dose of 25.5 GBq, which is the preferential dosage in tolerated patients.

Since the physical properties of ^90^Y suggest that this radionuclide will be more effective in larger tumor masses and ^177^Lu in smaller tumor masses and metastases, the combination treatment of ^90^Y- and ^177^Lu-labeled SST analogs seems a reasonable option for managing tumors of varying sizes and SSTR subtypes. As expected, both preclinical and clinical researches have found higher tumor response rate through the combined therapy [83, 86]. Nevertheless, the optimal combination of two radiopharmaceuticals should be determined on a patient-specific basis. As discussed by a recent article [90], the absorbed dose to tumors shows huge interpatient variance, and renal toxicity should be particularly considered since the biologically effective dose to the kidneys of ^177^Lu was lower compared with ^90^Y.

### 3.4. Studies with *α*-Emitters

**Figure 4:**
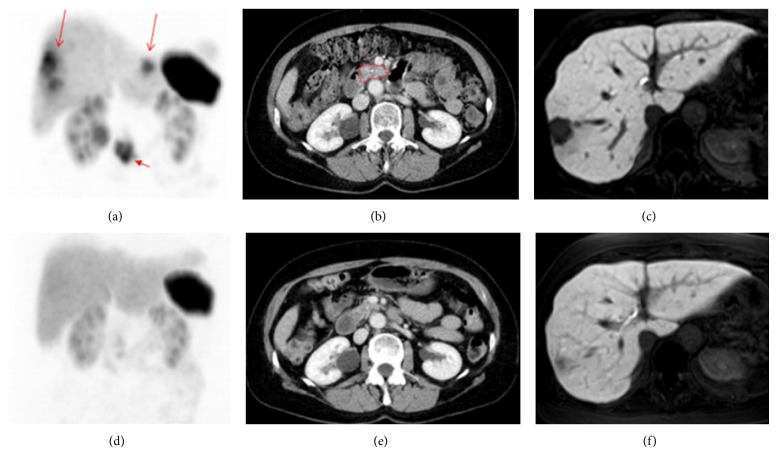
Patient 5 before therapy ((a)–(c)) and after three cycles of ^213^Bi-DOTATOC ((d)–(f)) to a dose of 4 GBq. (a) Beta-resistant residuals in the liver (long arrows) and primary tumor (short arrow) are present in the ^68^Ga-DOTATOC PET maximum intensity projection image. (b) Contrast enhanced CT image with the primary tumor outlined in red. (c) In the MR image with hepatocyte-specific contrast medium, the liver metastases appear as black cavities against the enhancing normal liver parenchyma. ((d)–(f)) After three cycles of ^213^Bi-DOTATOC to a dose of 4 GBq, the lesions have diminished on the PET image (d) and CT image (e). Also on the MR image (f), the residual lesion has almost disappeared as shown by the growth of normal hepatocytes demonstrated by the uptake of the hepatocyte-specific contrast medium [36].

The application of *α*-emitters such as ^213^Bi or its mother radionuclide ^225^Ac is arousing immense interest in PRRT. These radionuclides emit higher energy (8.32 MeV for ^213^Bi and 27.5 MeV for ^225^Ac) compared with *β*-emitters and had a short path-length of only 40–50 *μ*m, which increases the local antitumor effect without affecting untargeted tissues [90]. Alpha radiation can cause double-strand DNA breaks, independent of the cell cycle phase and oxygen concentration [91, 92]. Although PRRT with ^90^Y- and ^177^Lu-labeled SST analogs has been promising for NET therapy, some tumors are not radiosensitive to this treatment. SST analogs labeled with *α*-emitting isotopes may provide an alternative therapy for metastatic, chemoresistant, and hypoxic NETs, which are known to be resistant to PRRT with *β*-emitting radionuclides. A number of preclinical studies [93–95] have shown the potency and limited toxicity of targeted *α* therapy in NETs, while clinical trials were seldom studied. Recently, for the first time ^213^Bi-DOTATOC was used to treat patients with metastatic NETs refractory to therapy with ^90^Y/^177^Lu-DOTATOC [36]. Enduring responses were observed in all treated patients during follow-up for more than 2 years (Figure 4; Table 1). The side effects only include moderate chronic kidneys toxicity and favorable acute haematotoxicity. Nevertheless, *α* radiation with high linear energy transfer may lead to various and less repairable clustered damage, which may further induce secondary neoplasm formation [96]. Also it is unclear whether *α*-emitting radionuclides are superior to *β*-emitting radionuclides. Therefore, the therapeutic effects and safety should be further confirmed before *α* therapy can be well translated to clinical application.

## 4. Gene Transfection with hSSTr2

As previously presented, SSTR based imaging and therapy have made a great contribution to the diagnosis and treatment of NETs. However, many SSTR negative or weakly positive tumors, like non-small-cell lung cancer (NSCLC) [20], ovarian cancer [97], malignant glioma [98], and pancreatic cancer [18], are facing a big challenge in therapy. Surgery, chemotherapy, radiotherapy, or the combined therapy modalities have limited effects on improving overall survival. Most patients diagnosed with these tumors will ultimately suffer from recurrent diseases, resist further treatment, and eventually die of their diseases [99].

Over the past two decades, gene therapy has been applied in a number of malignant tumors and appears to be a safe and effective method for treatment. The TK gene, which is a suicide gene from herpes simplex virus (HSV), was widely studied. When HSV-TK is transfected into tumor cells in combination with intravenous ganciclovir, the antitumor efficacy is achieved through converting ganciclovir into a triphosphate configuration, which inhibits DNA synthesis and induces cell apoptosis [100]. Although noninvasive imaging of transferred gene expression has proven available following vector-mediated transfer of the HSV-TK using radiolabeled tracers, some studies [98, 101] found that hSSTr2 reporter based imaging was more sensitive and the uptake of radiolabeled SST analogs well correlated with recombinant vector dose. Moreover, SST analogs labeled with therapeutic radionuclides can be specifically delivered to transfected tumors, which provides an alternative approach to conventional therapeutics for SSTR negative or weakly positive tumors [102]. Since SSTR2 has been known to be most commonly expressed in various NETs and possesses the highest affinity for natural SST and synthetic SST analogs, most experiments utilize hSSTr2 reporter gene to transfect targeted tumor cells alone or together with other therapeutic genes.

In order to improve hSSTr2 gene transfer efficiency, it is critical to choose a vector with powerful infectivity. So far, different vector systems, mainly including adenovirus (Ad), retrovirus, adenoassociated virus, and vaccinia virus, have been employed in various tumor models [98, 109]. Ad remains the most frequently used and most promising virus for gene delivery because it has many advantageous features such as keeping itself stability, acquiring high titers, infecting a wide range of dividing cells as well as nondividing cells, permitting the high level expression of transferred gene, and maintaining a clear separation between viral genome and host chromosomes [103, 111]. Yet, a shortage that Ad depends on the coxsackie Ad receptor (CAR) to enter cells dramatically affects the transfection efficiency because many primary tumors do not express CAR [112]. Various approaches like genetic, chemical, and nonchemical modifications have been taken to retarget Ad vectors to other receptors [104]. The advantages and disadvantages of other main viral vectors used to transfer hSSTr2 are presented in Table 2.

## 5. Somatostatin Receptor Based Reporter Gene Imaging

**Figure 5:**
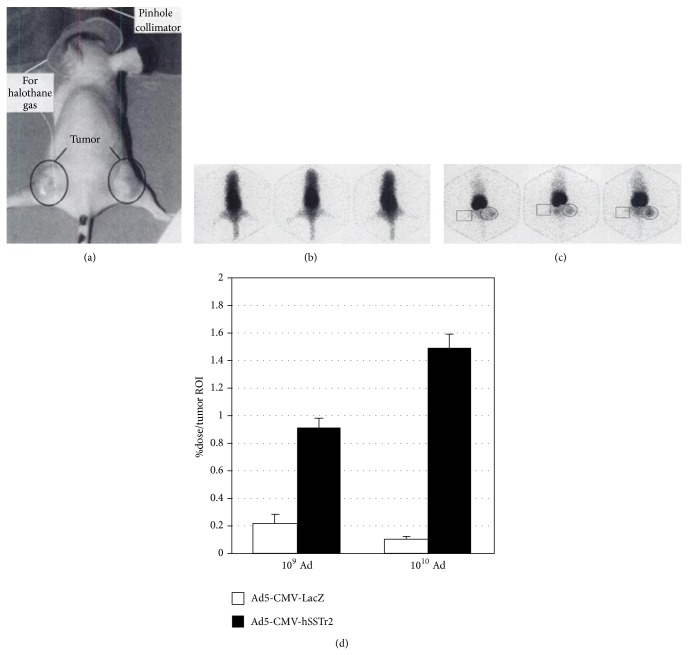
*γ* camera imaging of mice injected with ^99m^Tc-labeled SST analogs. Imaging position is shown in (a), with 3 representative images of mice at 3 minutes (b) and 3 hours (c) after intravenous injection. Circles in (c) indicate location of human A427 tumors injected 48 h earlier with Ad5-CMV-hSSTr2, and squares indicate human A427 tumors injected 48 h earlier with Ad5-CMV-LacZ. (d) Results of region of interest analyses [19]. A427 tumors: human non-small-cell lung cancer, hSSTr2 negative Ad5-CMV-LacZ: Ad encoding* Escherichia coli β*-galactosidase under control of the CMV promoter element.

Although gene therapy in various animal models has acquired encouraging results, many obstacles should be overcome before gene therapy can be well translated to clinical trials [113]. One obstacle is how to make sure that gene transfer occurs in targeted tissues. Some studies have tried to use optical imaging and biopsy to detect the expression levels of transferred genes. However, poor tissue penetration of light-based reporter systems [20] and invasive damage of biopsies [28] hamper a comprehensive assessment of the magnitude and time course of gene expression. Nuclear imaging based on hSSTr2 reporter gene transfer can get rid of these limitations. The hSSTr2 based reporter system has been utilized in a variety of tumor studies both* in vitro* and* in vivo* to estimate its ability to image gene transfer. A study [20] hoped to assess whether hSSTr2 is competent to act as a reporter of gene transfer. It used Ad encoding hemagglutinin A and SSTR2 (Ad-CMV-HA-hSSTr2) or control virus to transfect NSCLC cell lines and tumors bearing nude mice. As a result, the radiopeptide [^111^In-DTPA^0^]octreotide could specifically bind to tumor cells after Ad-CMV-HA-hSSTr2 transfection and the uptake of tracers in Ad-CMV-HA-hSSTr2 transfected tumors was higher than that of control groups. Similar results were reported by an article [19] which observed the expression of hSSTr2 originating from Ad5-mediated gene transfer to non-small-cell lung tumors with ^99m^Tc- or ^88^Re-labeled peptides (Figure 5). The article even calculated the approximate number of SSTR2 expressed per Ad5-transfected cell through the uptake of radiolabeled peptides, which provided more accurate information of gene expression. Except NSCLC, hSSTr2 reporter system was adopted to monitor the duration and time course of gene expression in other transfected tumors, including ovarian cancer [24], malignant glioma [98], and fibroblastoma [114], as well as normal tissues like muscle and liver [30]. All these laboratory results were so encouraging that hSSTr2 reporter gene system was applied to clinical trials. Kim et al. conducted a phase I clinical trial of Ad5.SSTR/TK.RGD in patients with recurrent gynecologic cancer [99]. The Ad vector not only contains the therapeutic gene TK but also contains the hSSTr2 reporter gene which allows for noninvasive and repetitive gene transfer imaging with [^111^In-DTPA^0^]octreotide. Compared to imaging before Ad-mediated gene therapy, significantly increased uptake of [^111^In-DTPA^0^]octreotide was found in patients after the last administration in the highest Ad dose group. All these studies indicate that the hSSTr2 reporter based imaging is a promising method to track gene delivery and expression. The detailed functions of hSSTr2 reporter gene based imaging are demonstrated below: repetitively observing the magnitude, duration, and time variation of gene expression both* in vitro* and* in vivo *[30], studying the biodistribution of gene transfer vector in mice or patients [25], optimizing the administration dose of vector encoding hSSTr2 reporter gene and/or other therapeutic genes [24], predicting treatment response of transfected tumor to PRRT [21], and monitoring antitumor effects of various treatments including hSSTr2 gene or another therapeutic gene based therapy [21, 99].

## 6. Somatostatin Receptor Based Reporter Gene Therapy

In addition to reporter based imaging, the hSSTr2 can serve as a therapeutic gene to cause tumor regression alone or together with other treatments [115]. As mentioned above, the prognosis of many malignant tumors is poor no matter what therapeutic methods are given. It is badly in need of new therapeutic approaches to treating these tumors. Impressive response of NET leading to improved survival has been observed with PRRT [116], which, however, is only suitable for tumors showing SSTR expression, but not for SSTR negative tumors. Fortunately, it is feasible to induce SSTR negative tumors to express SSTRs by means of gene transfer technology, which provides a novel therapy for some malignant tumors [22, 98, 117].

### 6.1. Therapy Studies with the Vector Encoding the Single hSSTr2 Gene

In a therapeutic study [22], Zhao et al. evaluated the antitumor effects of ^188^Re-RC-160 (^188^Re-labeled SST analog) on A549 tumor, which is one kind of lung adenocarcinomas, transfected with plasmid pcDNA3 encoding hSSTr2 reporter gene. Nude mice bearing pcDNA3-hSSTr2 transfected tumors were divided into five groups according to different therapeutic protocols. Finally, the tumor growth inhibition in the single dose treatment group (7.4 MBq, ^188^Re-RC-160) was significantly higher than that in ^188^Re group (2 × 7.4 MBq), RC-160 group, and saline control group. Moreover, two-injection group (2 × 7.4 MBq, ^188^Re-RC-160) led to significantly increased tumor growth inhibition compared with the single dose treatment group. These results provided a preliminary proof that SSTR negative tumor could be transfected with hSSTr2 reporter gene for radionuclide therapy. One problem observed in the present study was the low transfection efficiency. In fact, viral vectors have become the major vehicle to deliver hSSTr2 reporter gene. Although the drawbacks of viral vectors, especially immune response, are inevitable, transfection rate is fairly higher than that of nonviral vehicles and gene expression within tumors is relatively stable [118].

Reconstructed Ad encoding hSSTr2 gene (Ad-hSSTr2) was utilized in a study [119] to explore the therapeutic effects of [^90^Y]-SMT 487 ([^90^Y]-DOTA-_D_-Phe^1^-Tyr^3^-octreotide) on transfected tumors. Mice bearing non-small-cell lung tumors were intratumorally injected two doses of Ad-hSSTr2 (1 week apart) and intravenously administrated four doses of 14.8 MBq [^90^Y]-SMT 487 or four doses of 18.5 MBq [^90^Y]-SMT 487 with median tumor quadrupling times of respective 40 and 44 days, while in untreated group and the group that received only four doses of 18.5 MBq [^90^Y]-SMT 487 without virus, the median tumor quadrupling times were 16 and 25 days, respectively. Significant difference in time to tumor quadrupling between the groups that received Ad-CMV-hSSTr2 plus [^90^Y]-SMT 487 and the control groups was revealed by the log-rank test. The hSSTr2/[^90^Y]-SMT 487 system is a potential approach for clinical application, since both Ad vector encoding hSSTr2 gene and [^90^Y]-SMT 487 have been used for cancer therapy in clinical trials.

### 6.2. Therapy Studies with the Bicistronic Vector Encoding Both hSSTr2 and CD Genes

Gene therapy vectors containing both hSSTr2 reporter gene and a second therapeutic gene encoding TK or CD have been investigated [21, 99]. The hSSTr2 is available not only for noninvasive imaging of the expression of transferred gene, but also for radionuclide therapy [21]. A synergistic therapeutic effect may be achieved through the combined gene therapy. In view of the toxicity that resulted from the combination treatment of radiolabeled peptide and TK gene [34], the hSSTr2/CD system received considerable attention. Mechanisms of CD gene based therapy are that CD specifically converts the prodrug 5-FC to the highly toxic 5-FU, which disturbs DNA synthesis and induces cell death [120].

NSCLC A549 cells transfected by the bicistronic plasmid pCD-IRES-hSSTR2 (pCIS) were induced to express both SSTR2 and CD. Then 3 × 10^6^ pCIS-A549 cells were injected subcutaneously into each nude mouse on the right axilla and the same number of control A549 cells on the contralateral axillary regions of the same mouse to establish a xenograft tumor model. When tumors grew to an average diameter of 1cm, mice (*n* = 6) were intravenously injected with ^99m^Tc-RC-160, which specifically bound to pCIS-A549 cell-derived tumors. To study a synergistic inhibitory effect on tumor growth, ^131^I-RC-160 and 5-FC were injected alone or together into mice bearing tumors via their tail veins. The results showed that the combination treatment of those two agents inhibited pCIS-A549 cell-derived tumor growth significantly more than ^131^I-RC-160 or 5-FC treatment alone did [21]. These findings suggest that hSSTr2 reporter based therapy can combine with prodrug gene therapy to achieve enhanced antitumor effect and provides a novel treatment for lung cancer.

## 7. Conclusion

At present there are a number of tracers available for NET imaging. Their uptake is dependent upon different biological mechanisms, predominantly the expressions of SSTRs on tumor cell membranes. SRS with [^111^In-DTPA^0^]octreotide has played an important role in the diagnosis and staging of NETs. With the advent of PET technique, positron-emitting tracers were developed and seem to be more encouraging. ^68^Ga-DOTA-peptides used for PET or PET/CT imaging are hopeful of being routinely utilized to visualize NETs. PRRT is a most promising therapy for patients with inoperable and/or metastasized NETs. Treatment with ^90^Y-DOTATOC and ^177^Lu-DOTATATE has been shown to be relatively safe, and most patients had high objective tumor response after the therapy, especially the combined treatment of the two radiopeptides. In addition, hSSTr2 reporter gene based imaging and therapy are feasible in SSTR negative or weakly positive tumors by means of gene transfer technique. Radiolabeled SST analogs can be delivered to transfected tumors, which provides a new specific approach to imaging gene expressions and killing tumor cells. Furthermore, a synergistic therapeutic effect can be achievable by dual gene transfer of hSSTr2 reporter gene and a second therapeutic gene such as TK or CD gene. Though excellent results have been achieved with regard to hSSTr2 reporter gene based imaging and radionuclide therapy in SSTR negative or weakly positive tumors, additional preclinical and especially translational and clinical researches are needed to obtain further proof of value.

## Figures and Tables

**Table 1:** Tumor responses in patients with various NETs, treated with different radiolabeled SST analogs.

Ligand	Patient number	Tumor response				References
		CR	PR	SD	PD	
[^111^In-DTPA^0^]octreotide	26	0	2 (8%)	21 (81%)	3 (11%)	[71]
[^111^In-DTPA^0^]octreotide	18	0	2 (11%)	16 (89%)	0	[72]
^90^Y-DOTATOC	116	5 (4%)	26 (22%)	72 (62%)	13 (11%)	[79]
^90^Y-DOTATOC	41	1 (2%)	9 (22%)	25 (61%)	6 (15%)	[80]
^177^Lu-DOTATATE	310	5 (2%)	86 (28%)	158 (51%)	61 (20%)	[81]
^177^Lu-DOTATATE	26	3 (12%)	7 (27%)	12 (46%)	4 (15%)	[82]
^90^Y-DOTATATE and ^177^Lu-DOTATATE	26	2 (7.7%)	9 (34.6%)	11 (42.3%)	4 (15.4%)	[83]
^213^Bi-DOTATOC	7	1 (14%)	2 (28%)	3 (44%)	n.a.	[36]

CR: complete remission; PR: partial remission; SD: stable disease; PD: progressive disease; n.a. not available.

**Table 2:** The advantages and disadvantages of main viral vectors used to transfer hSSTr2.

Vectors	Advantages	Disadvantages	References
Adenovirus	(1) Stability (2) High titers (3) Infecting dividing and nondividing cells (4) High level expression of transgene (5) Not integrating into host chromosome	(1) Strong immune response (2) Potential replication competence (3) Short-term expression (4) Demanding packaging cell line (5) Small insert size (6) No targeting	[103–105]

Adenoassociated virus	(1) No associated disease (2) Long-term gene expression (3) Integrating into human chromosome 19	(1) Extensive antiviral immunity (2) Helper-dependent replication (3) Poor host tropism (4) Small insert size: about 5 kb	[106]

Retrovirus	(1) Integrating into host cell genome (2) Reverse transcription of the RNA genome (3) Infecting dividing cells (4) Long-term expression (5) Fairly high titers	(1) Immune-related toxicity (2) Infecting dividing cells (3) Potential replication competence (4) Insertion mutation (5) No targeting	[107, 108]

Vaccinia virus	(1) Cytolytic viral vector (2) Preferentially infecting rapid dividing cells (3) Difficult to leak from normal vasculature (4) The vector itself serving as a therapeutic method (5) Large insert size: ≥25 kb DNA	(1) Live infectious lytic virus (2) Replication competence (3) Short-term gene expression (4) Postvaccinal encephalitis and progressive complications (5) No targeting	[109, 110]

## Abbreviations

SST: Somatostatin
SSTR: Somatostatin receptor
SSTR2: Somatostatin receptor subtype 2
NETs: Neuroendocrine tumors
hSSTr2: Human somatostatin receptor subtype 2
GEP-NET: Gastroenteropancreatic neuroendocrine tumor
SRS: Somatostatin receptor scintigraphy
PET: Positron emission tomography
PRRT: Peptide receptor radionuclide therapy
SPECT: Single photon computed emission tomography
TK: Thymidine kinase
CD: Cytosine deaminase
CT: Computed tomography
MRI: Magnetic resonance imaging
WB DWI: Whole-body diffusion-weighted MR imaging
APUD: Amine precursor uptake and decarboxylation
CR: Complete remission
PR: Partial remission
SD: Stable disease
PD: Progressive disease
NSCLC: Non-small-cell lung cancer
HSV: Herpes simplex virus
Ad: Adenovirus
CAR: Coxsackie adenovirus receptor.
